# 107例*KRAS*突变阳性非小细胞肺癌患者临床分析

**DOI:** 10.3779/j.issn.1009-3419.2016.05.02

**Published:** 2016-05-20

**Authors:** 权 张, 敬慧 王, 曦 李, 卉 张, 靖颖 农, 娜 秦, 新勇 张, 羽华 吴, 新杰 杨, 嘉林 吕, 树才 张

**Affiliations:** 101149 北京，首都医科大学附属北京胸科医院，北京市结核病胸部肿瘤研究所，肿瘤内科 Department of Medical Oncology, Beijing Chest Hospital, Capital Medical University, Beijing Tuberculosis and Thoracic Tumor Research Institute, Beijing 101149, China

**Keywords:** *KRAS*突变, 肺肿瘤, 一线化疗, 靶向治疗, *KRAS* mutation, Lung neoplasms, First-line chemotherapy, Targeted therapy

## Abstract

**背景与目的:**

鼠类肉瘤病毒癌基因（Kirsten rat sarcoma viral oncogene, *KRAS*）是非小细胞肺癌（non-small cell lung cancer, NSCLC）的重要驱动基因之一，研究显示KRAS是表皮生长因子受体酪氨酸激酶抑制剂（epidermal growth factor receptor tyrosine kinase inhibitors, EGFR-TKIs）药物的耐药标志，但其对于化疗敏感性及预后方面的意义存在争议。本研究旨在积累KRAS突变阳性的NSCLC患者治疗经验。

**方法:**

我们回顾性分析了107例*KRAS*突变阳性的NSCLC患者的临床资料，分析*KRAS*突变阳性的NSCLC患者一线化疗疗效以及靶向治疗疗效。

**结果:**

52例接受一线化疗的晚期*KRAS*突变阳性NSCLC患者客观缓解率（objective response rate, ORR）为9.6%，疾病控制率（disease control rate, DCR）为53.8%，中位疾病无进展生存期（progression-free survival, PFS）为3个月；21例接受EGFR-TKIs药物治疗的*KRAS*突变阳性NSCLC患者ORR为9.5%，DCR为23.8%，PFS为1个月，其中*EGFR*/*KRAS*共突变患者接受EGFR-TKIs治疗的ORR及DCR均要显著高于单纯*KRAS*突变人群（50% *vs* 0, *P*=0.029; 75% *vs* 11.8%, *P*=0.043），*EGFR*/*KRAS*共突变患者接受EGFR-TKIs治疗的PFS较单纯*KRAS*突变患者延长，可见统计学差异（3个月*vs* 1个月，*P*=0.004）。

**结论:**

*KRAS*突变阳性NSCLC患者化疗有效率低，缓解时间短，EGFR-TKIs治疗效果差，亟需研发新的药物；*EGFR*/*KRAS*共突变现象客观存在，EGFR-TKIs药物可作为这类患者有效的治疗选择之一。

## Abstract (en)

**Background and objective:**

Kirsten rat sarcoma viral oncogene (*KRAS*) mutation is one of the major driver genes of non-small cell lung cancer (NSCLC). KRAS is a resistant predictor of epidermal growth factor receptor tyrosine kinase inhibitors (EGFR-TKIs), which raises controversy because of its role in chemotherapy sensitivity and prognosis. The aim of this study is to accumulate clinical experience in treating NSCLC patients harboring *KRAS* mutation.

**Methods:**

A total of 107 NSCLC patients harboring *KRAS* mutation were analyzed retrospectively. The efficacy was analyzed in terms of first-line chemotherapy or EGFR-TKIs therapy.

**Results:**

The objective response rate (ORR) to first-line chemotherapy of 52 patients with advanced disease harboring *KRAS* mutation was 9.6%. The disease control rate (DCR) was 53.8%, and the median progression-free survival (PFS) was 3 months. The ORR to EGFR-TKIs therapy in 21 patients harboring *KRAS* mutation and *EGFR*/*KRAS* co-mutation was 9.5%; the DCR was 23.8%, and the median PFS was 1 month. The ORR and DCR to EGFR-TKIs therapy of patients with *EGFR*/*KRAS* co-mutation were significantly higher than those of patients with *KRAS* mutation (50% *vs* 0, *P*=0.029; 75% *vs* 11.8%, *P*=0.043); the median PFS was also significantly longer (3 months *vs* 1 month, *P*=0.004).

**Conclusion:**

The efficacy to first-line chemotherapy and EGFR-TKIs therapy in NSCLC patients harboring *KRAS* mutation was poor; thus, new drugs should be developed. Furthermore, the existence of *EGFR*/*KRAS* co-mutation was confirmed. Hence, EGFR-TKIs therapy should be administered to patients with *EGFR*/*KRAS* co-mutation.

《2015年中国癌症登记统计》显示肺癌在我国恶性肿瘤发病率和死亡率均居于首位^[1]^。其中非小细胞肺癌（non-small cell lung cancer, NSCLC）占所有肺癌的80%左右。鼠类肉瘤病毒癌基因（Kirsten rat sarcoma viral oncogene, *KRAS*）是原癌基因RAS家族（KRAS, NRAS, HRAS）的成员之一，在肺癌信号传导级联中起核心作用的RAS/RAF/MEK/MAPK通路中*KRAS*突变最常见。LCMC的研究^[2]^显示肺腺癌*KRAS*驱动基因发生率为25%；而国内数据^[3-6]^显示肺腺癌*KARS*突变率为7.2%-8%，肺鳞癌*KRAS*突变率为5%。尽管不同种族人群*KRAS*基因的发生率不尽相同，但多项研究^[7, 8]^提示*KRAS*突变是NSCLC生存的不利影响因素，甚至有研究结果^[7]^显示*KRAS*突变的亚裔患者预后较非亚裔患者更差。我们对107例*KRAS*突变阳性NSCLC患者的临床数据进行回顾性分析，旨在为*KRAS*阳性NSCLC患者积累治疗经验。

## 1 资料与方法

### 1.1 纳入标准

2011年1月-2015年12月期间于首都医科大学附属北京胸科医院经组织学或胸水沉渣病理确诊为NSCLC的患者，经突变富集液相芯片法或扩增突变阻滞系统（amplification refractory mutation system, ARMS）基因检测结果显示为KRAS基因突变阳性或*EGFR*、*KRAS*基因共突变阳性。临床资料包括年龄、性别、吸烟状态、体力状况评分（performance status, PS）、病理类型、治疗方案等。肿瘤-淋巴结-转移（tumor-node-metastasis, TNM）分期以美国癌症联合会（American Joint Committee for Cancer, AJCC）第七版分期系统为标准。

### 1.2 排除标准

缺少*EGFR*、*KRAS*任一基因检测结果；缺少可评价病灶；5年内出现过或当前患有其他恶性肿瘤；存在任何重度和/或未能控制的心肌梗死、脑血管意外等其他疾病患者；具有精神类药物滥用且无法戒除或有精神障碍者；存在主要脏器功能严重障碍的患者。

### 1.3 治疗方案

包括手术治疗、术后辅助化疗、单药化疗、含铂两药联合方案全身化疗以及表皮生长因子受体酪氨酸激酶抑制剂（epidermal growth factor receptor tyrosine kinase inhibitors, EGFR-TKIs）靶向治疗。含铂两药联合方案包括：紫杉醇175 mg/m^2^，第1天，或长春瑞滨25 mg/m^2^，第1、8天，或吉西他滨1, 000 mg/m^2^，第1、8天，或培美曲塞500 mg/m^2^，第1天，或多西他赛75 mg/m^2^，第1天，联合顺铂75 mg/m^2^，第1天，或卡铂曲线下面积（area under the curve, AUC）5，第1天。上述联合方案21天为1周期。治疗过程常规应用预防性止吐及预处理措施。EGFR-TKIs靶向治疗包括吉非替尼、厄洛替尼以及埃克替尼。

### 1.4 评价标准及随访

采用实体瘤疗效评价标准评价疗效（Response Evaluation Criteria in Solid Tumors, RECIST）1.1版，疗效判定包括完全缓解（complete response, CR）、部分缓解（partial response, PR）、疾病稳定（stable disease, SD）及疾病进展（progression disease, PD）。客观缓解率（objective response rate, ORR）包括CR和PR。疾病控制率（disease control rate, DCR）包括CR、PR和SD。每2周期化疗后行计算机断层扫描（computed tomography, CT）影像学检查进行疗效评价，若1周期治疗后临床考虑疾病进展需经CT确认，EGFR-TKI靶向治疗以接受治疗1个月后行CT影像学检查进行疗效评价，记录以随访截止日期内最佳疗效为准。疾病无进展生存期（progression-free survival, PFS）从治疗第一天至疾病进展，或无疾病进展死亡的时间。随访截止日期为2015年12月30日。

### 1.5 统计学方法

应用SPSS 19.0统计软件进行数据处理及统计分析，计数资料比较采用卡方检验，采用*Kaplan*-*Meier*法计算患者的PFS，并绘制生存曲线。采用*Log*-*rank*检验分析各种因素对生存期的影响，以*P* < 0.05为差异有统计学意义。

## 2 结果

### 2.1 一般资料

符合纳入标准和排除标准的NSCLC病例共计107例，*KRAS*突变类型包括2号外显子第12位、第13位密码子突变及3号外显子第61位密码子突变，分别占88.8%、6.5%及3.7%，其中单纯*KRAS*突变阳性患者101例，*EGFR*、*KRAS*基因共突变阳性患者6例（19外显子缺失突变5例，21外显子L858R突变1例），56例患者曾接受手术治疗，52例复发或转移的晚期患者曾接受一线化疗，21例患者曾接受EGFR-TKIs药物治疗（表 1）。

**1:** 107例*KRAS*突变和*KRAS*/*EGFR*共突变NSCLC患者的基线临床特征 Baseline clinical characteristics of 107 NSCLC patients with *KRAS* mutation and *KRAS*/*EGFR* co-mutation

Characteristic	Data
Age (yr)	
Median	62
Range	20-81
Gender	
Male	74 (69.2%)
Female	33 (30.8%)
Smoking status	
Non-smoking	42 (39.3%)
Smoking	65 (60.7%)
Tumor histological type	
Squamous	10 (9.3%)
Adenocarcinoma	96 (89.7%)
Adenosquamouscarcinoma	1 (0.9%)
PS Scoring	
0	15 (14%)
1	90 (84.1%)
2	2 (1.9%)
TNM stage	
Ⅰ	10 (9.3%)
Ⅱ	10 (9.3%)
Ⅲ	30 (28.1%)
Ⅳ	56 (52.4%)
Unclear	1 (0.9%)
Gene mutation	
*KRAS* mutation	101 (94.4%)
*EGFR*/*KRAS* co-mutation	6 (5.6%)
Therapy with surgery	
Yes	56 (52.3%)
No	51 (47.7%)
Therapy with first-line chemotherapy	
Yes	52 (48.6%)
No	55 (51.4%)
Therapy with EGFR-TKIs	
Yes	21 (19.6%)
No	86 (80.4%)
EGFR-TKIs: epidermal growth factor receptor tyrosine kinase inhibitors; NSCLC: non-small cell lung cancer; KRAS: Kirsten rat sarcoma viral oncogene; TNM: tumor-node-metastasis; PS: performance status.	

### 2.2 一线化疗疗效

随访截止2015年12月30日，52例患者曾接受单药或含铂两药联合方案一线治疗，其中CR 0例，PR 5例，SD 23例，PD 24例，ORR为9.6%，DCR为53.8%，一线化疗PFS为3.0个月。对各临床特征同化疗疗效的关系进行统计学分析，未见统计学差异，通过对各临床特征、不同化疗方案之间一线化疗PFS进行统计学分析，仍然未见统计学差异（表 2）。

**2:** 52例接受一线化疗的患者临床特征与ORR/DCR/PFS的关系 Relationship between clinical characteristic and ORR/DCR to first-line therapy and relationship between clinical characteristic and PFS in 52 patients

Characteristic	*n*	ORR	*P*	DCR	*P*	Median PFS (range)	*P*
Gender			0.820		0.686		0.45
Male	34	11.8%		55.9%		3.6 (1.9-5.3)	
Female	18	5.6%		50.0%		2.0 (0.8-3.2)	
Age (yr)			0.999		0.366		0.965
< 60	29	10.3%		48.3%		2.5 (1.4-3.6)	
≥60	23	8.7%		60.9%		3.5 (2.6-4.4)	
Smoking status			0.500		0.829		0.805
Non-smoking	23	4.3%		52.2%		3 (1.9-4.1)	
Smoking	29	13.8%		55.2%		3.2 (1.1-5.3)	
PS scoring			0.999		0.208		0.107
0-1	50	10%		56%		3.0 (1.7-4.2)	
> 1	2	0		0		0.8 (0.8-2.5)	
TNM stage			0.999		0.495		0.450
Ⅲb	4	0		25%		1 (1.0-1.8)	
Ⅳ	48	10.4%		56.3%		3.0 (1.9-4.1)	
Tumor histological type			0.999		0.999		0.968
Adenocarcinoma	45	8.9%		53.3%		3.0 (2.1-3.9)	
Non-adenocarcinoma	7	14.3%		57.1%		2.4 (0-6.0)	
First-line chemotherapy regimen			0.127		0.482		0.413
With pemetrexed	20	20.0%		60.0%		3.6 (0.9-6.3)	
Without pemetrexed	32	3.1%		50.0%		3.0 (1.9-4.1)	
Total	52	9.6%		53.8%		3.0	
ORR: objective response rate; DCR: disease control rate.							

### 2.3 EGFR-TKIs靶向治疗疗效

随访截止2015年12月30日，21例患者曾接受EGFR-TKIs药物治疗，大多数患者因PS评分较差或患者、家属强烈要求而尝试采用该治疗方法，其中17例患者为单纯*KRAS*突变，其余4例为*EGFR*/*KRAS*共突变。*EGFR*/*KRAS*共突变患者中有2例疗效可达PR，*EGFR*/*KRAS*共突变患者接受EGFR-TKIs药物治疗的ORR及DCR均要高于单纯*KRAS*突变人群（*P*=0.029, *P*=0.043），*EGFR*/*KRAS*共突变患者接受EGFR-TKIs治疗的PFS较单纯*KRAS*突变患者延长，可见统计学差异（*P*=0.004）（表 3，图 1）。

**3:** 21例接受EGFR-TKIs治疗的患者临床特征与ORR/DCR/PFS的关系 Relationship between clinical characteristic and ORR/DCR to EGFR-TKIs therapy and relationship between clinical characteristic and PFS in 21 patients

Characteristic	*n*	ORR	*P*	DCR	*P*	PFS (mo)	*P*
Gender			0.133		0.530		0.085
Male	13	0		15.4%		1	
Female	8	25%		37.5%		1	
Age (yr)			0.999		0.935		0.391
< 60	15	6.7%		20%		1	
≥60	6	16.7%		33.3%		1	
Smoking status			0.214		0.903		0.159
Non-smoking	10	20%		30.0%		1	
Smoking	11	0		18.2%		1	
PS scoring			0.071		0.115		0.025
0-1	6	33.3%		50%		1	
2-4	15	0		13.3%		1	
TNM stage			0.649		0.999		0.661
Ⅲb	3	33.3%		33.3%		1	
Ⅳ	18	5.6%		22.2%		1	
Tumor histological type			0.999		0.532		0.285
Adenocarcinoma	17	11.8%		29.4%		1	
Non-adenocarcinoma	4	0		0		1	
Gene mutation			0.029		0.043		0.004
*KRAS*	17	0		11.8%		1	
*KRAS*/*EGFR* co-mutation	4	50%		75%		3	
Total	21	9.5%		23.8%		1	

**1:**
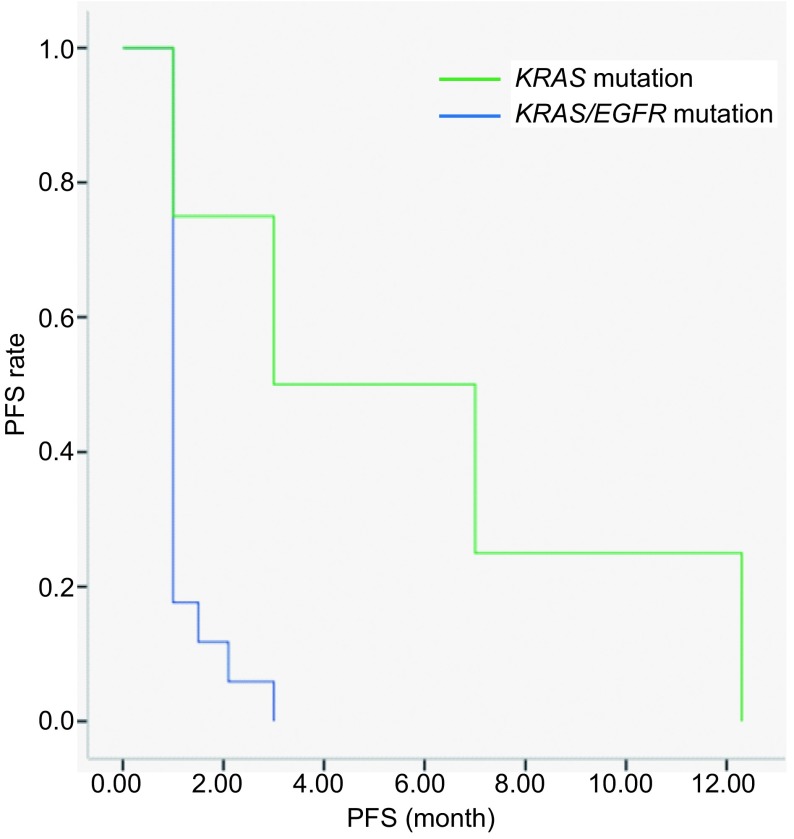
不同基因突变状态患者接受TKI药物治疗后PFS的差异 Progression-free survival (PFS) of patients according to different gene mutation

## 3 讨论

本研究共入组107例NSCLC患者，临床特征分析显示*KRAS*突变患者中位年龄为62岁，多见于男性及吸烟患者，与以往Xu等^[3]^研究及Wang等^[4]^相似。Xu等^[3]^对861例腺癌患者研究显示*KRAS*突变男性（51/493）多于女性（18/368），吸烟者（48/411）多于不吸烟者（20/439），Wang等^[4]^对332例腺癌患者研究也显示男性*KRAS*突变率（10.1%, 17/168）高于女性（4.3%, 7/164），吸烟患者*KRAS*突变率（11.7%, 14/120）高于非吸烟患者（4.7%, 10/212）。近些年来，对于多驱动基因发生共突变的现象已屡有报道，Bar等^[9]^研究显示8.3%的NSCLC患者肿瘤组织中存在2种或者2种以上的驱动基因突变，在本研究107例*KRAS*突变阳性患者中，存在5.6%（6/107）的*KRAS*/*EGFR*共突变。

本研究中共有52例患者接受一线化疗，ORR仅为9.6%，DCR为53.8%。既往多项研究分析了*KRAS*突变对于一线化疗的影响，结果仍有一定争议。Dong等^[10]^研究EGFR、KRAS对于NSCLC一线化疗缓解率的影响，其中携带KRAS突变病例共计64例，接受含吉西他滨或多西他赛或长春瑞滨方案进行一线化疗，ORR和DCR高达31.9%和81.3%；Kalikaki等^[11]^对162例局部晚期或晚期接受化疗的NSCLC患者进行回顾性分析，*KRAS*突变患者一线化疗ORR为26.5%，较野生型未见明显差异（25.0%, *P*=0.087）。Mellema等^[12]^开展的一项回顾性研究结果证实*KRAS*突变对晚期非鳞肺癌一线化疗疗效无预测作用，*KRAS*突变与野生型患者的ORR无差别（*P*=0.77）。而Campos-Parra等^[13]^研究显示*KRAS*突变患者一线化疗有效率为14.7%，低于野生型患者（41.4%, *P*=0.03）。本组KRAS患者患者一线化疗中位PFS为3.0个月，较Campos-Parra等^[13]^研究结果类似（3.3个月）。仅为非选择晚期NSCLC患者3个月-5个月的一线化疗PFS的下限，*KARS*突变患者一线化疗缓解时间短。2013年发表的一项*meta*分析^[7]^，纳入41项研究共6, 939例NSCLC患者分析KRAS的预后价值，结果显示，*KRAS*突变是NSCLC生存的不利影响因素（HR=1.45, 95%CI: 1.29-1.62），分层分析显示，*KRAS*突变的亚裔患者的预后较非亚裔患者更差，HR分别为1.97（95%CI: 1.58-2.44）和1.37（95%CI: 1.25-1.5），*KRAS*突变是亚裔患者预后差的标志。本研究中*KRAS*突变组ORR较低，可能与病例数较少，且化疗方案相对较分散有关，其中含培美曲塞方案相对其他化疗方案缓解率似乎略有优势，但未见统计学差异，也未观察到PFS获益，仍需开展大样本临床研究探索KRAS阳性患者一线化疗的最佳方案或是研发新的药物或治疗办法。

目前已有多项研究^[7, 14-16]^证实*KRAS*突变患者对EGFR-TKIs药物原发耐药，*KRAS*突变患者EGFR-TKI疗效有效率不足3%，低于野生型患者。Metro等^[17]^的回顾性研究结果显示，接受EGFR-TKIs治疗的*KRAS*突变患者的PFS仅有1.6个月，与本研究结果类似。而对于*EGFR*/*KRAS*共突变的患者人群，EGFR-TKIs药物可作为有效的治疗选择之一，Benesova等^[18]^研究显示在15例*KRAS*突变阳性人群中发现3例*EGFR* 19缺失突变，应用吉非替尼治疗后疗效均可达PR，Choughule等^[19]^研究发现4例*EGFR*/*KRAS*共突变患者，*EGFR*突变类型包括19外显子缺失突变及21外显子L858R突变，接受EGFR-TKIs后肿瘤均得到有效缓解。本研究中共计发现6例*EGFR*/*KRAS*共突变患者，4例接受EGFR-TKIs治疗，2例获得PR，1例为SD，1例无效，该EGFR-TKIs治疗无效病例病理类型为鳞癌，DCR的差异最终转化为PFS获益，达3个月。因此，对于*EGFR*/*KRAS*共突变患者，不可错失EGFR-TKIs药物治疗的机会。

本研究为回顾性研究，样本量有限，早些年相关基因检测送检率低，可能存在一定的抽样误差，各组患者接受治疗的种类及方案不均一，不足以分析不同治疗方案之间的差异，部分患者后续随访中出现失访，尚不能有效总结*KRAS*突变对DFS以及OS的影响，仍有待后续前瞻性大样本临床研究提供更高级别的循证医学证据。

综上所述，*KRAS*突变患者化疗有效率低，EGFR-TKIs治疗效果差，需开展更多研究探索更为有效的治疗手段和临床药物；*EGFR*/*KRAS*共突变现象客观存在，该类患者应考虑EGFR-TKIs治疗。
